# Feasibility of upright carbon ion radiotherapy for prostate cancer: Dosimetric comparison between supine and upright postures

**DOI:** 10.1002/mp.70555

**Published:** 2026-07-13

**Authors:** Yusuke Nomura, Taku Inaniwa, Yoshitake Yamada, Minoru Yamada, Yoichi Yokoyama, Hideyuki Mizuno, Shunsuke Yonai, Yoshiyuki Iwata, Kohei Oguma, Junichi Fukada, Naoyuki Shigematsu, Atsuya Takeda, Hitoshi Ishikawa, Masahiro Jinzaki

**Affiliations:** ^1^ Department of Accelerator and Medical Physics, Institute for Quantum Medical Science National Institutes for Quantum Science and Technology Chiba Japan; ^2^ Department of Radiology Keio University School of Medicine Tokyo Japan; ^3^ QST Hospital National Institutes for Quantum Science and Technology Chiba Japan

**Keywords:** carbon ion radiotherapy, prostate cancer, upright treatment

## Abstract

**Background:**

Carbon ion radiotherapy (CIRT) in the upright posture is a treatment technique which delivers carbon ion beams from a fixed direction to patients who sit or stand on a rotating chair. Although various potential advantages of the upright positioning have been discussed, feasibility of upright CIRT for prostate cancer has not been quantitatively evaluated.

**Purpose:**

This study demonstrated prostate cancer CIRT in the upright posture and evaluated its feasibility by comparing anatomy and dosimetric metrics between the supine and upright postures.

**Methods:**

A total of 12 pairs of computed tomography (CT) images of asymptomatic volunteers in the supine and upright postures were retrospectively analyzed. Based on a clinical treatment protocol, clinical target volume (CTV) was defined as being a prostate gland and proximal seminal vesicle. Other target and organ‐at‐risk (OAR) contours within a planning volume were also delineated. A CIRT plan was calculated for each CT volume with the same dose prescription and evaluation criteria. The delineated contour volumes, 16 dosimetric metrics, dose‐volume histograms (DVHs) of CTV and OARs, and robustness against setup and range uncertainties were compared between the supine and upright postures.

**Results:**

No significant differences in the contour volumes were found between the two postures (*p* > 0.05). The CTV and rectum contour volumes were 34.30 ± 7.76 and 36.35 ± 13.53 cm^3^ for the supine posture and 35.89 ± 7.37 and 32.96 ± 8.08 cm^3^ for the upright posture, respectively. Moreover, mean absolute differences of five CTV dosimetric metrics (D2%, D5%, D50%, D95%, and D98%) were all less than 0.15 Gy (0.29% of the prescription dose of 51.6 Gy). Nine other dosimetric metrics in the upright posture were also equivalent to those in the supine posture. The DVHs and robustness in the upright posture were in agreement with those in the supine posture.

**Conclusions:**

The upright CIRT provided dose distributions with target dose coverage and OAR dose sparing equivalent to those of the conventional supine CIRT. Upright CIRT for prostate cancer is expected to have equivalent treatment effectiveness while offering reduced installation costs and simple system management of platforms.

## 1 INTRODUCTION

Carbon ion radiotherapy (CIRT) is an advanced radiotherapy technique delivering a highly conformal dose to targets while sparing an unnecessary dose to normal tissues. Since CIRT provides a localized dose with high linear energy transfer, numerous studies have reported favorable treatment outcomes and controlled toxicity for various complex and radioresistant cancers at prostate,^1^, ^2^ lung,^3^ pancreas,^4^ head and neck,^5^ and other body sites. Despite these promising results, the numbers of available CIRT platforms are much lower than those of photon or proton radiotherapy mainly because of a large treatment vault space, high construction cost, and complicated system management. For example, a rotating gantry in CIRT is much larger and more expensive than that in the other beam types of radiotherapy.^6^, ^7^

One potential solution for these issues is conducting CIRT in the upright posture.^8^, ^9^ Various studies^10^, ^11^, ^12^ have discussed possible advantages of upright radiotherapy over conventional supine radiotherapy. For instance, the upright CIRT platforms can be installed in smaller vault spaces than supine CIRT platforms by utilizing a rotating chair instead of the gantry to deliver beams to patients over one rotation. Management of the upright platforms also becomes simpler for various reasons, including the fact that the rotating chair does not use complicated beam control devices.^10^, ^12^ Since 13 out of 17 (76%) CIRT facilities worldwide do not have the gantry but at least one horizontal beam port (last update, April 2026),^13^ the upright radiotherapy will be useful for these facilities to expand available beam angles with smaller vault space and lower costs. Moreover, these advantages are also beneficial for new facilities which are considering to install new CIRT platforms.

To clinically conduct upright CIRT while utilizing these advantages effectively, feasibility of the upright CIRT needs to be evaluated against the conventional supine CIRT. While various cancers and diseases have been treated with CIRT, 40% of the treated patients worldwide were genitourinary or prostate cancer patients, and this percentage is the largest among other types of cancer.^14^ Therefore, feasibility analysis of upright CIRT for prostate cancer is crucial for clinical applications. However, very few studies have investigated upright CIRT for prostate cancer. Schreuder et al.^11^ reported only anatomical differences in the pelvis of healthy volunteers between the supine and upright postures without any dosimetric analysis. Oguma et al.^15^ showed that the upright photon dose distributions were equivalent to supine dose distributions, and differences in all dosimetric metrics for rectum and bladder were within ± 1.4%. However, the treatment planning in CIRT, including contour delineation, margin settings, beam angles, and dose calculation methods, is different from that in photon radiotherapy. Therefore, these differences justify the importance of investigating the anatomical and dosimetric changes in upright CIRT.

This study aims to demonstrate upright CIRT for prostate cancer and evaluate its feasibility by comparing anatomy and dose distributions between the supine and upright postures. Supine and upright computed tomography (CT) images of the same subjects were acquired on the same day^16^, ^17^, ^18^ and were utilized to assess anatomical changes and dose distributions. While the methodology of this study was comparable to the authors’ previous work for pancreatic cancer,^19^ this study investigated upright CIRT for prostate cancer. To the best of the authors’ knowledge, this is the first study to quantitatively evaluate feasibility of the upright CIRT for prostate cancer in comparison with the conventional supine CIRT.

## 2 METHODS

### 2.1 CT image acquisition

CT images from 100 healthy participants in the supine and upright (standing) postures from a previous study^17^ were retrospectively analyzed. From these data, 12 male subjects were randomly selected; they were 32–68 years old (mean: 49.2 years) and weighed 61.4–88.0 kg (mean: 74.3 kg). The supine CT images were acquired with a conventional CT scanner (Aquilion ONE, Canon Medical Systems, Otawara, Japan). The upright CT images were acquired with an upright CT scanner (prototype TSX‐401R, Canon Medical Systems).^16^, ^18^ Image resolution was 0.976 mm × 0.976 mm × 0.5 mm in both supine and upright CT images. The average scan time interval between these scans over all subjects was 104 min (range: 14–160 min). To reproduce clinical planning CT images at National Institutes for Quantum Science and Technology (QST), the CT images were downsampled from the slice thickness of 0.5 mm to 2.0 mm.

The procedures for CT imaging and its analysis were approved by the Keio University School of Medicine Ethics Committee (No. 20221150) and the QST Institutional Review Board (No. N22‐103).

### 2.2 Target and organ delineation and treatment planning

**FIGURE 1:**
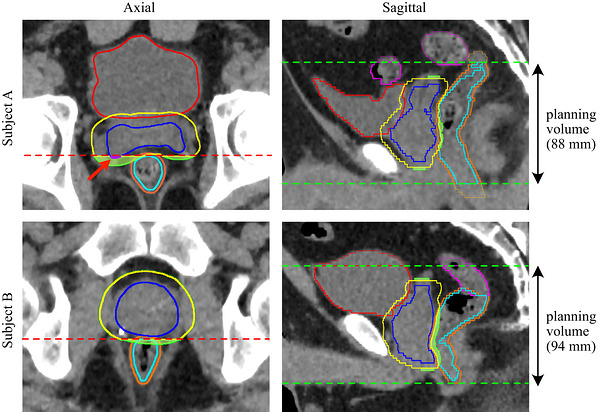
Axial and sagittal CT images of two representative subjects with delineated contours. Red and green dotted lines represent a lateral cut line passing through the anterior edge of the rectum‐2mm contour and planning volume boundary, respectively. The OAR contours outside the planning volume were also delineated with thin solid lines for visualization purposes. The purple area pointed out by a red arrow indicates the trimmed CTV volume with the lateral cut line. The green area is the subtracted volume of the PTV2 from the PTV1. Blue = CTV, green = PTV1, yellow = PTV2, orange = rectum, cyan = rectum‐2mm, red = bladder, magenta = sigmoid colon.

Target and OAR contours were generated by using image processing software, MIM Maestro version 7.3.3 (MIM Software Inc., Cleveland, OH, USA). The contour definition and delineation procedure were followed by the clinical protocol.^1^, ^2^, ^20^ All contours were manually delineated or reviewed by an experienced radiation therapist and a radiation oncologist. The delineated target and OAR contours of representative subjects are illustrated in Figure 1. Clinical target volume (CTV) was defined as being the prostate gland and a proximal seminal vesicle. The OARs were comprised of the rectum, sigmoid colon, intestine, and bladder. The CTV and OAR contours, as well as the rectum contour with 2 mm negative margins (contour shrinkage) along the left‐right and anterior‐posterior directions, denoted as the “rectum‐2mm” contour, were delineated. To optimize treatment plans while meeting OAR dose constraints effectively, a lateral cut line passing through the anterior edges of the rectum‐2mm, sigmoid colon, and intestine contours were drawn for each axial slice (dotted red line in Figure 1), and the subvolume of the CTV posterior of the lateral cut lines (purple area) was trimmed out. Subsequently, the first planning target volume (PTV1) was made by expanding the CTV by 10 mm, 7 mm, and 5 mm along the lateral, anterior, and other directions, respectively. Moreover, the second PTV (PTV2) was generated by trimming out the PTV1 with the lateral cut lines and removing its top and bottom axial slices (green area). The target and OAR contours were delineated within the CT images ranging from 10 mm above the superior boundary of PTV1 to 10 mm below its inferior boundary. This delineation volume was referred to as a planning volume.

Carbon ion beam dose calculations and their optimization were performed with in‐house dose calculation software XiDose^21^, ^22^, ^23^ and its wrapper codes PyDOSE.^19^ The dose was represented as the product of the absorbed dose and clinical relative biological effectiveness (RBE) based on the microdosimetric kinetic model.^24^ A plan with two lateral opposing fields was optimized for each CT volume with single field uniform dose (SFUD) optimization. In accordance with the clinical protocol,^1^, ^2^, ^20^ the RBE‐weighted uniform dose of 51.6 Gy and 43.9 Gy was prescribed to the PTV2 and its subtracted volume from the PTV1 (green area in Figure 1) in 12 fractions, respectively. Clinical dose criteria used in treatment planning are listed in Table 1. The rectum constraints were obtained from a reference dose‐volume histogram (DVH) calculated by averaging the unnormalized DVHs of 51 prostate cancer patients with grade 0 rectal toxicity.^25^

**TABLE 1:** Clinical dose criteria for treatment planning. V*x* represents a volume receiving a dose greater than or equal to *x* Gy, and Dmax indicates the maximum dose.

	Criteria
Rectum	V40 ≦ 4.15 [cm^3^]
	V45 ≦ 2.57 [cm^3^]
	V50 ≦ 0.44 [cm^3^]
	Dmax ≦ 52.2 [Gy]
Sigmoid colon and intestine	Dmax ≦ 36.0 [Gy]
Whole body	Dmax ≦ 55.2 [Gy]

### 2.3 Evaluation of anatomy and dose distributions

Subject anatomical differences between the supine and upright postures were assessed by comparing volumes of the target and OAR contours. The PTV2 volumes can vary because the relative position of the proximal seminal vesicle against the prostate may change.^11^ The OAR contour volumes were compared as an indirect indicator of their positions relative to the CTV; a smaller OAR contour volume indicated that a larger OAR volume was located outside the planning volume. Overlapped volumes between the PTV1 and OAR contours were also compared to assess dose optimization complexity. Moreover, a difference in the volumes between these postures was calculated by subtracting the volume in the supine posture from that in the upright posture. A relative difference in the volumes was also obtained by dividing this difference by the volume in the supine posture.

To compare the optimized dose distributions between the supine and upright postures, 16 dosimetric metrics of CTV and OARs were calculated. The dose to target volumes was evaluated with the minimum doses received by 2%, 5%, 50%, 95%, and 98% of the CTV (D2%, D5%, D50%, D95%, and D98%), homogeneity index (HI), and conformity index (CI). The HI was defined as the ratio of the residual dose between CTV D5% and D95% to CTV D50%. The CI was derived from the ratio of the volume receiving more than 98% of the prescription dose to the PTV2 volume. For the OAR dose evaluation, volumes receiving at least 40 Gy, 45 Gy, and 50 Gy (V40, V45 and V50) for the rectum and the maximum dose (Dmax) to the whole body, rectum, sigmoid colon, and intestine were calculated in accordance with the clinical dose criteria for treatment planning (Table 1). V40 and V50 of the bladder were also obtained.

The dose distributions were further evaluated by comparing DVHs of the CTV and OARs. Because the uniform dose was prescribed to the CTV regardless of its volume, the normalized DVH of the CTV was compared. On the other hand, since the delineated OAR contour volumes were different between the postures, the unnormalized DVHs of OARs were compared.

To evaluate effects of the upright positioning on robustness of the dose delivery against uncertainty, a robustness analysis was performed. The variations of dose distributions due to the uncertainty were represented by recalculating the optimized plans for the CT images with simulated setup and range uncertainties. Based on previous studies about supine radiotherapy,^26^, ^27^, ^28^ the setup uncertainty was set to translational shifts of ± 3 mm or ± 5 mm along each 3D axis, while the range uncertainty was set to ± 3%. Hence, a total of 25 scenario dose distributions (i.e. 1 nominal scenario and 12 scenarios each with ± 3%/ ± 3 mm or ± 3%/ ± 5 mm uncertainties) were calculated for each plan. Since clinically realistic uncertainty in the upright posture could not be determined, the same uncertainties were simulated in supine and upright postures. The robustness was assessed by calculating proportions of the scenarios in which each of the eight robustness criteria was met, which are called acceptance rates. The robustness criterion for the target volume was that the CTV D95% must be at least 95% of the prescription dose. The robustness criteria for the OARs were the same as the clinical dose criteria (Table 1). The separately calculated acceptance rates for each criterion were compared between the postures.

All analyses and evaluations were performed with Python version 3.11.7 and Scipy version 1.11.4.^29^ Statistically significant differences were assessed with the Wilcoxon signed‐rank test and the Fligner–Killeen test for each comparison of contour volumes. The Mann‐Whitney U test was used for each comparison of dosimetric metrics and acceptance rates. A significance level of 0.05 was used for all tests.

## 3 RESULTS

### 3.1 Anatomical changes

**FIGURE 2:**
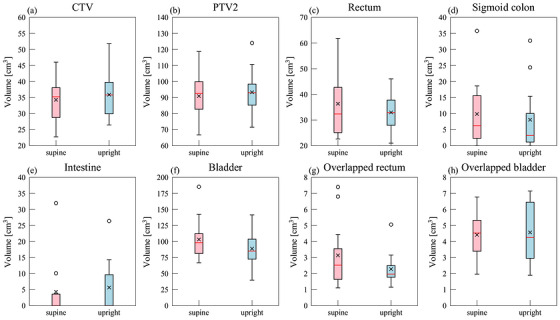
Comparison of the CTV, PTV2, and OAR contour volumes between the supine and upright postures. (a) and (b) show the CTV and PTV2 volumes, and (c), (d), (e), and (f) illustrate the rectum, sigmoid colon, intestine and bladder volumes, respectively. Overlapped volumes of the rectum and bladder with the PTV1 are also shown in (g) and (h), respectively. The bottom and top edges of the box plots represent the first and third quartiles of the data. The red lines indicate the median values. The whiskers are extended from the box edges to the maximum or minimum data point within 1.5 times the inter‐quartile range. The mean value and outliers are plotted by cross and circle marks, respectively.

**FIGURE 3:**
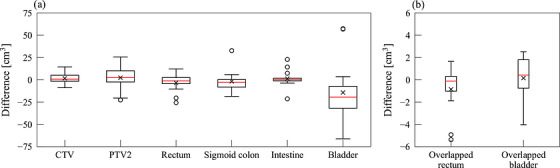
Difference in the contour volumes between the supine and upright postures. (a) shows the differences in the CTV, PTV2, and OAR volumes. (b) illustrates the differences in overlapped volumes of the rectum and bladder with PTV1. Positive values indicate that the volumes in the upright posture were larger than those in the supine posture.

Figure 2 illustrates the CTV, PTV2, OAR, and overlapped OAR volumes in the supine and upright postures. The overlapped volumes of the sigmoid colon and intestine are not shown because their volumes were almost zero for all subjects in both postures. Detailed volumes and *p* values are summarized in Table S1. All volumes did not have statistically significant differences between these postures (*p* > 0.05). The mean and standard deviation values of the CTV, PTV2, and rectum volumes for all subjects were 34.30 ± 7.76 cm^3^, 90.94 ± 15.76 cm^3^, and 36.35 ± 13.53 cm^3^ in the supine posture and 35.89 ± 7.37 cm^3^, 93.31 ± 14.81 cm^3^, and 32.96 ± 8.08 cm^3^ in the upright posture, respectively. Although not statistically significant (Fligner–Killeen test *p* = 0.066), the standard deviation of the overlapped rectum volumes decreased from 2.08 cm^3^ for the supine posture to 1.05 cm^3^ for the upright posture. Moreover, the volume differences between these postures are shown in Figure 3. Most of the differences were within ± 30 cm^3^ for targets and OARs. The mean relative differences in the CTV, PTV2, rectum, and bladder volumes for all subjects were 7.10%, 4.01%, −4.09%, and −7.32%, respectively. Differences in the overlapped OAR volumes were also close to zero for most subjects.

### 3.2 Dosimetric metrics and dose‐volume histograms

**FIGURE 4:**
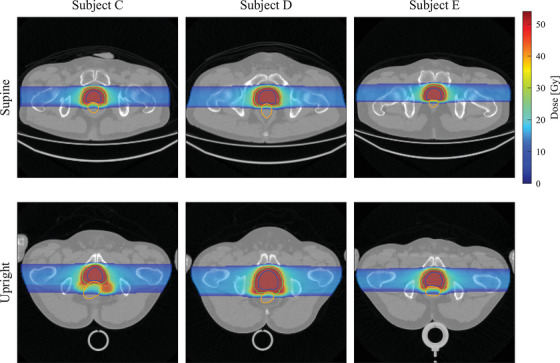
Dose distributions for three subjects in both the supine and upright postures. Blue, green, and orange lines represent the CTV, PTV1, and rectum contours, respectively.

Figure 4 shows the supine and upright dose distributions of three representative subjects. Both supine and upright dose distributions provided a highly conformal dose to the CTVs while sparing the dose to the rectums.

**FIGURE 5:**
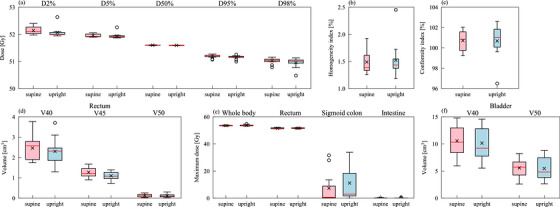
Comparison of dosimetric metrics between the supine and upright dose distributions. (a) illustrates D2%, D5%, D50%, D95%, and D98% of the CTV, (b) illustrates HI, and (c) illustrates CI. (d) shows V40, V45, and V50 of the rectum. (e) indicates the Dmax to the whole body, rectum, sigmoid colon, and intestine. (f) shows V40 and V50 of the bladder.

Comparison of the dosimetric metrics between the supine and upright postures (Figure 5) indicated that all metrics did not show statistically significant differences between these postures (*p* > 0.05). The mean absolute differences of CTV D2%, D5%, D50%, D95%, and D98% were 0.141 Gy, 0.068 Gy, 0.010 Gy, 0.054 Gy, and 0.112 Gy, respectively, showing an almost identical dose to the CTV in these dose distributions. This result was also supported by HI and CI which had mean and standard deviation values of 1.5% ± 0.2% and 100.7% ± 1.0% for the supine posture and 1.5% ± 0.3% and 100.7% ± 1.6% for the upright posture, respectively. Most upright OAR dosimetric metrics were also equivalent to the supine metrics except that the sigmoid colon Dmax was slightly higher in the upright posture than supine posture without statistical significance (*p* = 0.198).

**FIGURE 6:**
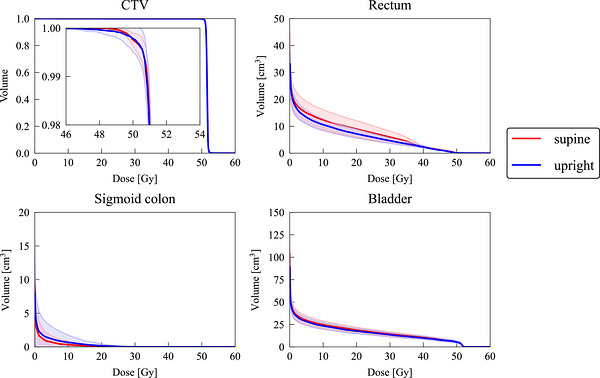
Comparison of DVHs for the CTV and OARs in the supine and upright postures. The solid line and shaded band represent mean and 95% confidence interval of DVHs, respectively. The OAR DVHs are not normalized by their volumes because the volumes differed between the postures.

**FIGURE 7:**
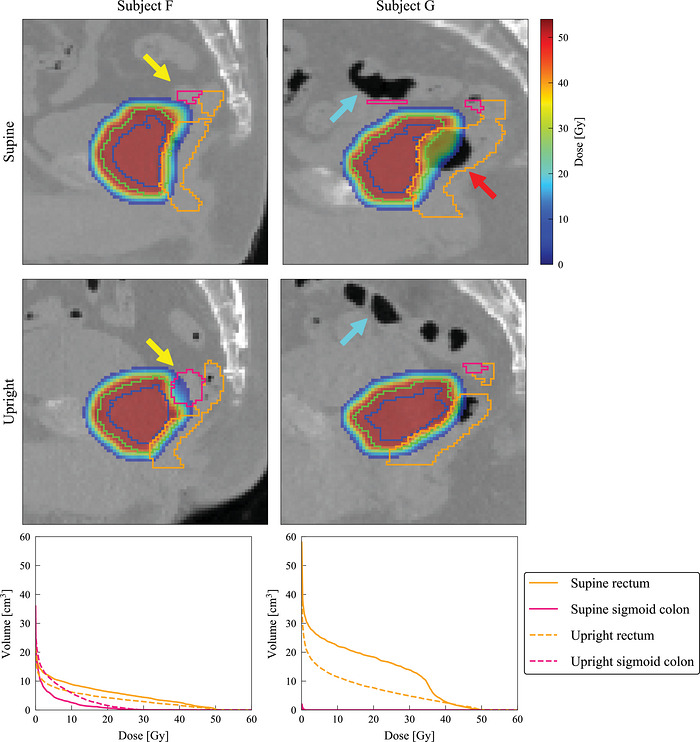
Comparison of dose distributions and DVHs of two representative subjects at the mid‐sagittal slices in the supine and upright postures. Blue, green, orange, and magenta lines represent the CTV, PTV1, rectum, and sigmoid colon contours, respectively. Subject F received a higher dose to the sigmoid colon in the upright posture than the supine posture, while subject G received a higher dose to the rectum in the supine posture than the upright posture. Yellow, red, and cyan arrows indicate remarkable anatomical changes in the sigmoid colon, rectum, and rectal gas, respectively.

The DVHs of the CTV and OARs are compared between the supine and upright postures in Figure 6. The CTV DVHs are normalized by their volumes, while the OAR DVHs are not normalized. The mean DVHs in the upright posture were well overlapped by those in the supine posture. The CTV DVHs in the upright posture were almost identical to those in the supine posture. The sigmoid colon and rectum DVHs had slightly larger and smaller variances in the upright posture than the supine posture, respectively. To further investigate possible reasons for these differences, the dose distributions at the mid‐sagittal slices passing through pubic symphysis and DVHs of a respective subject each with a large difference in sigmoid colon and rectum DVHs are illustrated in Figure 7. Subject F received a higher dose to the sigmoid colon in the upright posture than the supine posture because the sigmoid colon was shifted toward the inferior direction and became closer to the target (as pointed out by yellow arrows in Figure 7). Subject G received a higher dose to the rectum in the supine posture than the upright posture because the rectal gas moved to the anterior direction and pushed the anterior rectal wall (red arrow). Due to change in the gravity direction, gastrointestinal gas motion changed from the anterior direction in the supine posture to the superior direction in the upright posture (cyan arrows).

### 3.3 Robustness analysis

**FIGURE 8:**
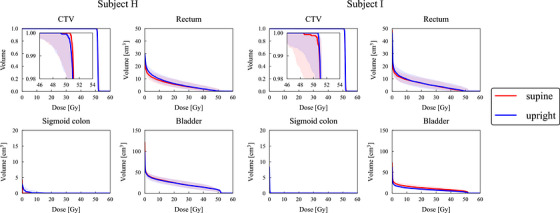
DVHs for the CTV and OARs of two representative subjects with ± 3%/ ± 3 mm uncertainties. A solid line and shaded band represent the nominal DVH and range of all scenario DVHs, respectively.

The CTV and OAR DVHs of two representative subjects with the simulated ± 3%/ ± 3 mm uncertainties are illustrated in Figure 8. The figure shows that the upright DVHs provided equivalent variations to the supine DVHs. Similar trends were also observed for the ten other subjects and the DVHs with the ± 3%/ ± 5 mm uncertainties. Comparison of the acceptance rates for all subjects between the postures (Table 2) shows that the upright CIRT had comparable robustness of the dose delivery to the supine CIRT. The degradation of the acceptance rates due to the increased setup uncertainty was also consistent between the postures. No statistically significant differences in the acceptance rates were found between the postures (*p* > 0.05).

**TABLE 2:** Comparison of the acceptance rates (mean ± standard deviation) for all subjects between the supine and upright postures.

	Criteria	Supine [%]		Upright [%]	
		±3%/ ± 3 mm	±3%/ ± 5 mm	±3%/ ± 3 mm	±3%/ ± 5 mm
CTV	D95% ≧ 95%	96.2 ± 6.1	83.3 ± 13.1	96.8 ± 7.7	89.1 ± 12.9
Rectum	V40 ≦ 4.15 [cm^3^]	70.5 ± 24.9	69.2 ± 22.7	78.2 ± 12.2	73.7 ± 6.9
	V45 ≦ 2.57 [cm^3^]	78.2 ± 7.9	75.0 ± 7.4	75.0 ± 9.9	70.5 ± 4.4
	V50 ≦ 0.44 [cm^3^]	57.7 ± 15.6	57.7 ± 14.5	65.4 ± 7.0	65.4 ± 7.0
	Dmax ≦ 52.2 [Gy]	40.4 ± 13.6	41.7 ± 14.1	47.4 ± 10.3	48.1 ± 10.4
Sigmoid colon	Dmax ≦ 36.0 [Gy]	93.6 ± 17.9	91.7 ± 17.8	90.4 ± 18.9	88.5 ± 18.7
Intestine	Dmax ≦ 36.0 [Gy]	100.0 ± 0.0	100.0 ± 0.0	100.0 ± 0.0	100.0 ± 0.0
Whole body	Dmax ≦ 55.2 [Gy]	100.0 ± 0.0	100.0 ± 0.0	100.0 ± 0.0	99.4 ± 2.2

## 4 DISCUSSION

This study demonstrated prostate cancer CIRT in the upright posture and compared its anatomy and dose distributions against those in the supine posture. Comparison of delineated contour volumes (Figure 2) revealed that the anatomical changes in target and OAR volumes due to the upright positioning were not statistically significant. The relative volume differences of the CTV, PTV2, rectum, and bladder contours were within ± 8% (Figure 3). Similar anatomical comparisons have also been made by Schreuder et al.^11^ and Oguma et al. .^15^ On the other hand, this study evaluated these anatomical differences in the context of CIRT dosimetric comparison. Moreover, the dosimetric metrics and DVHs showed that the upright CIRT provided a highly conformal dose to the CTV while sparing dose to the OARs similar to the conventional supine CIRT (Figure 5). While subject‐specific variations in the OAR doses were observed (Figure 7), the mean CTV and OAR DVHs were well overlapped between these postures. The robustness analysis also indicates that the upright CIRT provided comparable robustness against the uncertainties to the supine CIRT (Table 2). Consequently, the upright CIRT has great potential to treat prostate cancer using the gantry‐less platforms with reduced installation costs and simple system management.

While no statistically significant differences were found, the anatomical changes in the upright posture resulted in the increased sigmoid dose and the decreased rectum dose in a few subjects (Figure 7). Since the sigmoid colon shift varies among the subjects and its estimation is very difficult, image acquisition in the upright posture is crucial for selecting the appropriate treatment posture for each patient. Moreover, due to the change in gravity direction, the rectal gas moved superiorly in the upright posture. The rectal gas in the upright posture was seen to apply less pressure to the anterior rectum walls than that in the supine posture. These anatomical variations in the gravity‐induced rectal gas shift have also been observed in the upright posture by Oguma et al.^15^ and in the prone posture by Bentel at el.^30^ and Ikeda et al. .^31^ However, this study presents the first report about its effect on CIRT dose distributions. In the future, the effects of other pathological factors for the anatomical changes such as bladder filling or rectum content will be evaluated for more precise dose delivery in the upright posture. The possibility of intra‐fractional organ drifts due to the posture changes^10^, ^32^ will also be assessed.

The robustness analysis shows that the upright positioning did not largely affect the plan robustness (Figure 8 and Table 2). It should be noted that the acceptance rates in the upright posture might not be realistic because the setup and range uncertainties in the upright posture have not been obtained. However, since all scenarios were calculated with the same uncertainties in both postures, this analysis allows for fair comparisons of the robustness against simulated uncertainties between the postures. When the upright uncertainties are quantified, it will be possible to make a more realistic analysis in the future.

This study applied the downsampling of CT slice thickness from 0.5 mm to 2.0 mm to reproduce planning CT images and to perform clinically reasonable contour delineation. A previous study^33^ reported that the slice thickness was not associated with delineated contours, except that the bladder contour volume increased by thinning the slice thickness due to some potential factors such as a partial volume effect. These factors might also degrade the contour delineation accuracy in this study. However, since these effects occur in both supine and upright CT images in the same manner, the downsampling is not considered to largely affect the anatomical comparison. Moreover, the impact of downsampling on the dosimetric comparison is limited because the CIRT dose distributions were calculated with the same dose grid size (2.0 mm × 2.0 mm × 2.0 mm) in both postures.

A limitation of this study is that the delineated contours might not be accurate due to the limited expertise in the upright anatomy and the lack of magnetic resonance images which are often acquired along with CT images in clinics. Although the pelvic organs and their filling can move dynamically,^34^, ^35^ the supine and upright CT images could not also be acquired at the same time. These discrepancies may lead to inaccurate anatomical comparisons and dose calculations. Another limitation is that the CT images could not perfectly reproduce the characteristics of actual patients because scanned subjects were healthy volunteers and they were not instructed to drink water prior to the CT image acquisition. Therefore, whole bladder volumes in some CT images were not more than 100 mL in accordance with the clinical protocol.^1^, ^2^, ^20^ However, since Schreuder et al.^11^ reported that the prostate position and shape were not affected by the bladder filling in the upright posture, the full bladder preparation might not be needed in upright CIRT. It should be noted that the volumes of the bladder contours delineated within the planning volume (Figure 2) are not always identical to the whole bladder volumes. Moreover, a look‐up table which converts CT numbers into carbon stopping power ratio was not calibrated for the CT images due to the lack of calibration data in the previous study.^17^ In this study, a look‐up table calibrated for a different planning CT scanner from the same manufacturer was used, which might cause dose calculation error. In addition to these major limitations, subject arms in the upright CT images were ignored during the dose calculation because they were partially covered along the beam paths (Figure 4). Nevertheless, this issue can be readily addressed in practice by using an arm rest.^36^, ^37^ To address these limitations, further investigations and clinical trials using more CT images of actual patients will be conducted in the future.

The upright therapy will be a crucial CIRT technique because it allows for irradiating beams from any beam angles without using a rotating gantry.^6^, ^7^ Some previous studies^38^, ^39^ showed that treatment plans with anterior oblique beams reduced OAR doses and enhanced robustness against setup uncertainty. Therefore, upright CIRT will also be useful to increase the treatment efficacy by using these oblique beams. Feasibility of the oblique beam plans in the upright posture will be investigated. Moreover, the upright CIRT is effective due to its remarkable cost reduction and simple system management. When low‐cost and compact upright CIRT platforms are widely installed, more patients will be able to select the CIRT treatment. Since this study only examined the upright CIRT for prostate cancer, feasibility and clinical applications for various other cancers will also be investigated in the future.

## 5 CONCLUSIONS

Feasibility of the upright CIRT for prostate cancer was evaluated by comparing the pelvic anatomy and dose distributions between the supine and upright postures. The upright CIRT delivered a highly conformal dose equivalent to the supine CIRT. Since upright CIRT platforms with a rotating chair will provide technical and financial benefits over rotating gantry‐based supine platforms, the upright dose delivery is expected to become an advanced CIRT technique widely adopted in clinics.

## CONFLICT OF INTEREST STATEMENT

Masahiro Jinzaki received a grant from Canon Medical Systems, and the funder also lent an upright CT scanner to Keio University. All other authors have no conflict of interest to declare.

## Supporting information



Supporting information

## Data Availability

Within the bounds of reasonable requests, approvals by the Keio University School of Medicine and QST are needed to share data. The request applications are reviewed to check all ethical and research conditions.
