# Supplementary material for: Feasibility of upright carbon ion radiotherapy for prostate cancer: Dosimetric comparison between supine and upright postures
Source: Med Phys. 2026 Jul 13;53(7):e70555. doi: 10.1002/mp.70555 (PMC13361178; doi:10.1002/mp.70555)
Supplement: Supplementary file 1 — Supporting information [file MP-53-0-s001.pdf]

Supplementary Materials

Title: Feasibility of upright carbon ion radiotherapy for prostate cancer: Dosimetric comparison between supine and upright postures

Delineated contour volumes

Mean, standard deviation, and range of contour volumes are summarized in Table S1. The overlapped volumes of the sigmoid colon and intestine with the PTV1 are not listed because their volumes were almost zero for all subjects in both postures.

Table S1. Delineated contour volumes [cm^3] in the supine and upright positions. SD indicates standard deviation.

		Supine			Upright			Wilcoxon	Fligner-Killeen
		Mean	SD	Range	Mean	SD	Range	p value	p value
3D volume	CTV	34.30	7.76	22.77 - 46.06	35.89	7.37	26.45 - 51.80	0.380	0.793
	PTV1	100.46	17.80	69.82 - 129.63	99.92	13.67	81.38 - 130.81	0.970	0.645
	PTV2	90.94	15.76	66.75 - 118.74	93.31	14.81	71.50 - 124.02	0.569	0.832
	Rectum	36.35	13.53	22.61 - 61.85	32.96	8.08	20.97 - 46.07	0.733	0.262
	Sigmoid colon	9.88	10.47	0.00 - 35.79	8.09	10.61	0.00 - 32.75	0.266	0.494
	Intestine	4.33	9.21	0.00 - 31.98	5.67	8.37	0.00 - 26.41	0.735	0.732
	Bladder	103.20	33.90	66.97 - 185.39	88.94	29.23	39.86 - 141.63	0.151	0.923
Overlapped volume with PTV1	Rectum	3.14	2.08	1.11 - 7.40	2.28	1.05	1.15 - 5.06	0.424	0.066
	Bladder	4.42	1.56	1.97 - 6.78	4.57	1.84	1.89 - 7.15	0.569	0.353
